# Phosphosites of the yeast centrosome component Spc110 contribute to cell cycle progression and mitotic exit

**DOI:** 10.1242/bio.059565

**Published:** 2022-11-07

**Authors:** Marjan Abbasi, Alexander Julner, Yan Ting Lim, Tianyun Zhao, Radoslaw Mikolaj Sobota, Victoria Menéndez-Benito

**Affiliations:** ^1^ Department of Biosciences and Nutrition, Karolinska Institutet, SE-141 83 Huddinge, Sweden; ^2^ Functional Proteomics Laboratory, SingMass National Laboratory, Institute for Molecular and Cell Biology (IMCB), Agency for Science, Technology, and Research (A*Star), 138 673 Proteos, Singapore

**Keywords:** Microtubule, Mitotic spindle, Phosphorylation, Spindle pole body, Pericentrin, Cdc14

## Abstract

Spc110 is an essential component of the spindle pole body (SPB), the yeast equivalent of the centrosome, that recruits the γ-tubulin complex to the nuclear side of the SPB to produce the microtubules that form the mitotic spindle. Here, we identified phosphosites S11 and S36 in maternally originated Spc110 and explored their functions *in vivo*. Yeast expressing non-phosphorylatable Spc110^S11A^ had a distinct spindle phenotype characterised by higher levels of α-tubulin, which was frequently asymmetrically distributed between the two SPBs. Furthermore, expression of the double mutant Spc110^S11AS36A^ had a delayed cell cycle progression. Specifically, the final steps of mitosis were delayed in Spc110^S11AS36A^ cells, including expression and degradation of the mitotic cyclin Clb2, disassembling the mitotic spindle and re-localizing Cdc14 to the nucleoli, resulting in late mitotic exit and entry in G1. Thus, we propose that Spc110 phosphorylation at S11 and S36 is required to regulate timely cell cycle progression in budding yeast.

This article has an associated First Person interview with the first author of the paper.

## Teaser

**Summary:** We identified two phosphosites (S11 and S36) in the yeast centrosome component Spc110 from maternal origin and found that these phosphosites control timely cell cycle progression.

## INTRODUCTION

Centrosomes and yeast spindle pole bodies (SPBs) are known for their conserved roles in microtubule (MT) nucleation and spindle formation for chromosome segregation. In budding yeast, the SPB is a layered structure inserted in the nuclear envelope throughout mitosis (Cavanaugh and Jaspersen, 2017). The MTs are assembled by the γ-tubulin small complex (γ-TuSC), a conserved complex composed of γ-tubulin (Tub4), Spc97 and Spc98 in budding yeast. The γ-TuSC binds the SPB via two receptors: Spc72 on the outer plaque (cytoplasmic side), and Spc110 (the yeast homolog of pericentrin, PCNT) on the inner plaque (nuclear side) (Knop and Schiebel, 1998). The cytoplasmic MTs grow towards the cell cortex, while the nuclear MTs bind to kinetochores (kinetochore MTs) and grow towards the opposite SPB (interpolar MTs) meeting at the spindle midzone.

Moreover, centrosomes and SPBs serve as signalling platforms for cell cycle regulators, checkpoint proteins and MT-associated proteins, and have a direct role in cell cycle regulation (Arquint et al., 2014). For example, the budding yeast SPB acts as a regulatory hub for components of the mitotic exit network (MEN), a GTPase-driven signalling cascade that ultimately activates the protein phosphatase Cdc14, which is the major effector of mitotic exit (Baro et al., 2017). The MEN components – the GTPase Tem1, the protein kinase Cdc15 and the protein kinase complex Dbf2-Mob1 – localise to the cytoplasmic side of the SPBs. Tem1 is bound to the SPB component Nud1 during mitosis (Bardin et al., 2000; Molk et al., 2004; Pereira et al., 2000; Valerio-Santiago and Monje-Casas, 2011). In the mother cell, the protein complex Bub2-Bfa1 inactivates Tem1 (Pereira et al., 2000). By contrast, the Tem1 activator, Lte1, localises to the bud cortex, and thus Tem1 activation is coupled with SPB segregation to the bud (Bertazzi et al., 2011; Falk et al., 2011). Then, Tem1, together with the polo-kinase Cdc5, activates the protein kinase Cdc15 and docks it to the SPB (Rock and Amon, 2011; Visintin and Amon, 2001). In turn, Cdc15 phosphorylates Nud1, generating a docking site for Dbf2-Mob1 (Rock et al., 2013). Finally, Dbf2-Mob1 and Cdc5 promote the sustained release of Cdc14 from the nucleolus (Mah et al., 2001; Manzoni et al., 2010; Mohl et al., 2009; Shou et al., 1999), where it was bound to Cfi1/Net1 and kept inactive (Visintin et al., 1999). Cdc14 is partly released from the nucleolus at the metaphase to anaphase transition through the FEAR pathway (Stegmeier et al., 2002). In late anaphase, activation of MEN results in a sustained Cdc14 release that localises to the cytoplasm, the SPBs (Yoshida et al., 2002), and the bud neck, where it plays a role in the splitting of the septin ring and actomyosin contraction (Tamborrini et al., 2018).

Previous research has shown that all the SPB components are phosphoproteins. In total, 297 phosphosites have been identified by phosphoproteomic analysis from intact SPBs, and many of these sites are cell cycle specific (Fong et al., 2018; Keck et al., 2011). However, only a few SPB phosphosites have been functionally characterised so far. For example, phosphorylation of Sfi1 ensures that the SPB duplicates only once per cell cycle (Avena et al., 2014; Elserafy et al., 2014). Interestingly, the SPB duplication results in an old (from maternal origin) and a new SPB, which segregate in an age-dependent manner, where the old SPB migrates to the newly formed daughter cell (Pereira et al., 2001). Cell-cycle phosphorylation by the kinases Swe1, Kin3 and Cdc5 contributes to the age-determination of SPBs (Lengefeld et al., 2017; Matellán et al., 2020). Particularly, Swe1 phosphorylates the SPB outer plaque component Nud1 (Y40 /Y76) in G1, before the assembly of the new SPB, to mark the old SPB (Lengefeld et al., 2017). There may be other age specific SPB phosphorylation events that await further investigation.

The phosphorylation of Tub4, Spc72 and Spc110 that regulate MT nucleation are some of the best functionally characterised phosphosites of the SPB (Friedman et al., 2001; Huisman et al., 2007; Lin et al., 2011, 2014; Pereira et al., 1999; Vogel et al., 2001). Spc110 is phosphorylated in a cell cycle-dependent manner, starting at S phase, and accumulates during mitosis. The Mps1 (S60 T64 T68) and cyclin-dependent kinase 1, Cdk1, (S36 and S91) Spc110 phosphorylation sites stimulate γ -TuSC oligomerization at the SPB and activate MT nucleation (Lin et al., 2014). Blocking Spc110 phosphorylation at S91 causes a delay in metaphase (Huisman et al., 2007). Furthermore, loss of Cdk1-dependent Spc110 phosphorylation induces spindle polarity and stability defects (Huisman et al., 2007).

Experiments using fluorescence recovery after photobleaching (FRAP) have shown that Spc110 undergoes a dynamic exchange in and out of the SPB in G1/S until the spindle is fully assembled, when Spc110 becomes stable (Yoder et al., 2003). In a previous study, we followed the exchange of Spc110 in dividing cells using recombination-induced tag exchange (RITE) (Menendez-Benito et al., 2013). RITE is based on a genetic switching of epitope tags by an inducible Cre recombinase to differentially label old and newly synthesised proteins in budding yeast (Verzijlbergen et al., 2010). Using a RITE tagging cassette that switches between two fluorescent tags (GFP and mRFP), we analysed the distribution of old (from maternal origin) and newly synthesised Spc110 and found that at late anaphase Spc110 from maternal origin (GFP-tagged) was predominantly (90%) located at the bud SPB, while new (mRFP-tagged) Spc110 was incorporated into both SPBs (Menendez-Benito et al., 2013).

Here, we applied RITE to purify stable, old (from maternal origin) Spc110 for mass spectrometry analysis. Our studies identify two phosphosites in old Spc110 that regulate cell cycle progression for timely mitotic exit.

## RESULTS

### Purification of Spc110 from maternal origin from intact yeast SPBs

**Fig. 1.**
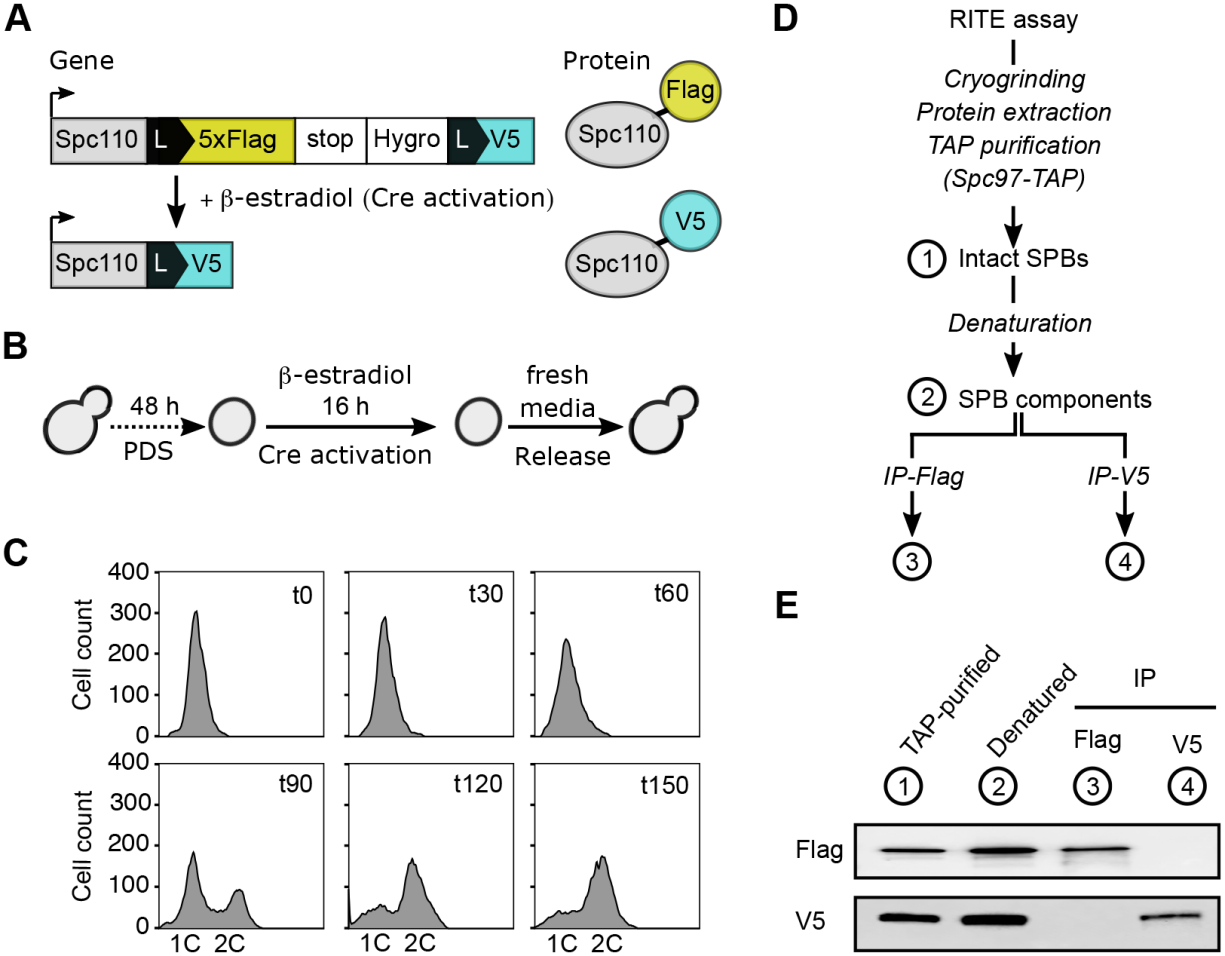
**Enrichment of Spc110 from maternal origin with Recombination-induced tag exchange (RITE).** (A) Outline of RITE. The upper panel shows a RITE cassette containing two epitope tags (5xFlag and V5) integrated downstream of *SPC110*. The first epitope tag (5xFlag) has a LoxP sequence at the 5′ end, and is followed by an ADH1 terminator sequence (stop), a Hygromycin resistance gene (Hygro), and a second LoxP sequence. The lower panel shows the RITE cassette after recombination. (B) Experimental procedure to coordinate RITE recombination and cell division. The cells are grown to the post-diauxic shift (PDS), and then Cre-recombinase is activated by adding β-estradiol to induce the tag exchange. Afterwards, the cells are released and start dividing, expressing new Spc110 (V5-tagged) and duplicating the SPB. (C) Cell cycle progression during the release from PDS, monitored by flow cytometry. Representative flow cytometry profiles of DNA content at different times (0-150 min) after release. One of three independent experiments is shown. (D) Experimental workflow to separate Spc110 from maternal origin and newly synthesised Spc110. After performing the RITE assay as shown in (B), cells were cryo-grinded, proteins extracted, and intact SPBs were purified by IgG-affinity purification of Spc97-TAP (1). Then, the SPB complexes were denatured to disrupt protein–protein interactions (2). Finally, maternal (Flag-tagged)- and newly synthesised (V5-tagged)- Spc110 proteins were purified by immunoprecipitation (IP) with antibodies against Flag (3) or V5 (4). (E) Western blot analyses of fractions from TAP-purification (1), denaturation (2), immunoprecipitation with Flag (3) or V5 (4) antibodies. The western blots were probed with anti-Flag (top) and anti-V5 (bottom) antibodies. One of three independent experiments is shown.

To explore the phosphosites of old (from maternal origin) Spc110, we generated a yeast RITE strain expressing epitope tags amenable for affinity-based purification. We used a yeast strain that constitutively expresses Cre-recombinase fused to the estrogen-binding domain (EBD), Cre-EBD78 previously described (Lindstrom and Gottschling, 2009), and integrated a RITE cassette behind the *SPC110* gene (Fig. 1A). The RITE cassette had an epitope tag (5xFlag), flanked by two LoxP recombination sites, followed by a second orphan epitope tag (V5). We can activate the Cre-EBD recombinase with β-estradiol, resulting in an epitope tag exchange to distinguish proteins made before (Flag) and after (V5) recombination.

To coordinate the epitope tag exchange with the SPB duplication, we used the RITE assay previously developed to follow SPB inheritance (Menendez-Benito et al., 2013) (Fig. 1B). We induced the genetic switch during the post-diauxic shift (PDS), which is a natural state when yeasts consume all the glucose in the media (Werner-Washburne et al., 1996). Inducing the genetic switch during the PDS, we achieve a high recombination efficiency (Fig. S1A-B), while cell proliferation and protein synthesis are minimal (Menendez-Benito et al., 2013). We then release the cells in media with glucose to induce cell cycle re-entry. To assess the synchrony of the cultures, we measured their DNA content by flow cytometry (Fig. 1C). Immediately before the release, there was a single 1C DNA peak indicating an efficient arrest by glucose depletion. A second peak of cells with 2C DNA content (G2/M) appeared 1.5 h later. The G2/M population increased and reached 80% of the cells after 2.5 h.

We next set up a protocol to purify Spc110-Flag and Spc110-V5 (Fig. 1D). We collected samples 2.5 h after the release and isolated intact SPBs by co-purification with the γ-TuSC component Spc97, as described by Fong et al. (2018). We then used trichloroacetic acid (TCA) precipitation and boiled the samples with SDS to break down the intact SPBs. Afterwards, we resuspended the proteins in a mild denaturing buffer, divided the sample into two and performed immunoprecipitations against Flag or V5. Our western blot analyses showed that Spc110-Flag (old) and Spc110-V5 (new) proteins were efficiently separated with no apparent cross-contamination (Fig. 1E).

### Identification of phosphosites (pS11, pS36 and pS60) in Spc110 from maternal origin

**Fig. 2.**
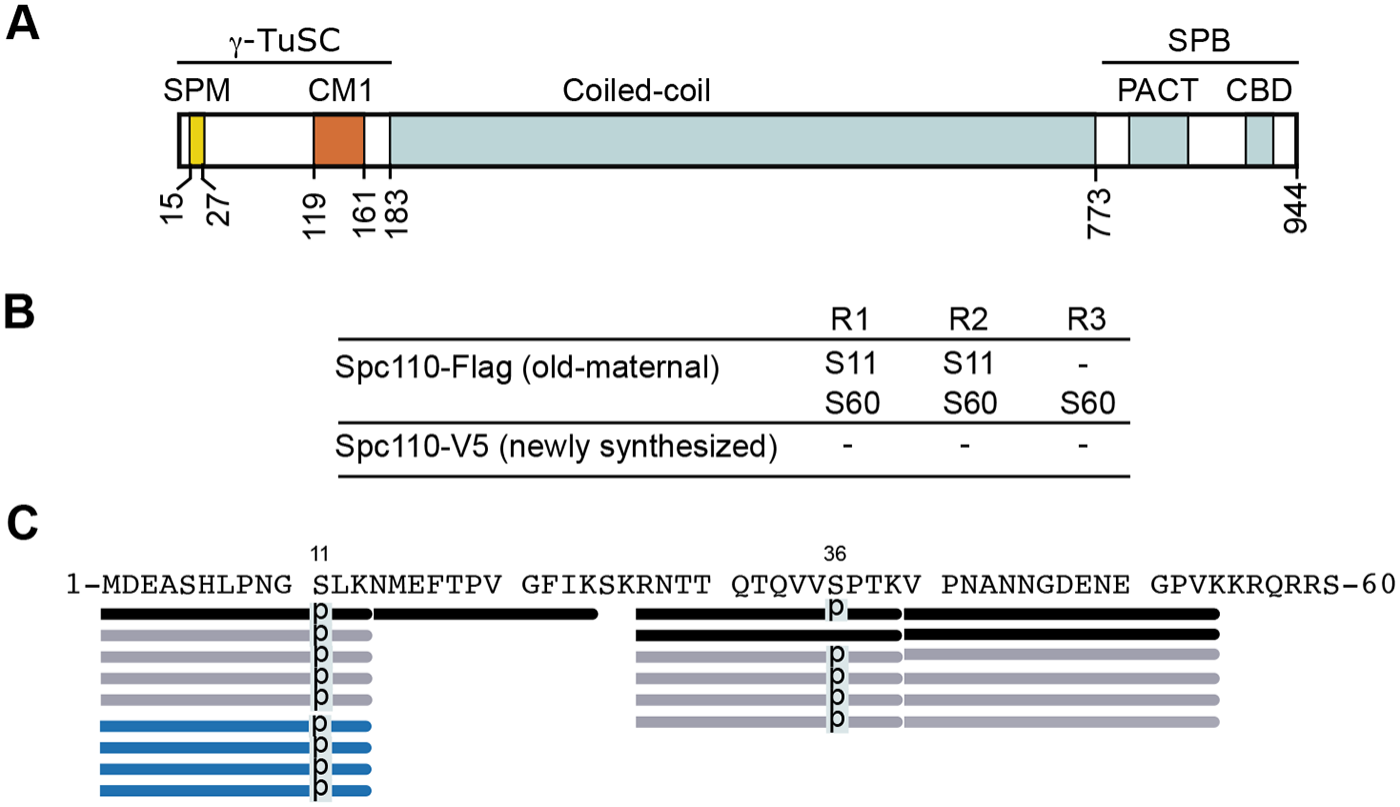
**Mass spectrometry analysis of Spc110 from maternal origin identifies phosphosites S11, S36 and S60.** (A) Spc110 functional domains and known interactions. Lines and numbers indicate residue boundaries of domains. γ-TuSC, γ-tubulin small complex; SPB, spindle pole body; SPM, Spc110/Pcp1 motif; CM, centrosomin motif 1; PACT, pericentrin-AKAP450 centrosomal targeting; CBD, calmodulin-binding domain. (B) Phosphosites identified after phospho-enrichment in Spc110-Flag (old, from maternal origin) and Spc110-V5 (newly synthesised) purified as shown in Fig. 1D in three biological replicates (R1-R3). (C) Phosphosites identified in Spc110-Flag (old, from maternal origin) without phospho-enrichment. Peptides (*P*<0.005) identified at the N-terminal sequence of Spc110 are represented with bars and phosphosites are indicated with ‘p’. Different colours represent data from each biological replicate (black=R1; grey=R2 and blue=R3) and phosphosites are indicated with a ‘p’.

We next purified Spc110-Flag (old, from maternal origin) and Spc110-V5 (newly synthesised) in three biological replicates, followed by mass spectrometry analyses. First, we enriched the purified samples for phospho-peptides using titanium dioxide (TiO_2_) beads. The MT nucleating Spc110 phosphorylation sites, the Mps1 (S60 T64 T68), and Cdk1 (S36 and S91) sites lie in the Spc110 N-terminal region (Fig. 2A). We found the Mps1 phosphosite pS60 in Spc110-Flag (Fig. 2B). Furthermore, we identified a phosphosite in pS11 in (old) Spc110-Flag. By contrast, we did not find phosphosites in Spc110-V5.

Next, we ran the purified samples by mass spectrometry without phospho-enrichment. Since we did not detect phosphosites in the Spc110-V5 (new) samples, we focused our analyses on Spc110-Flag (old, from maternal origin). We attained a sequence coverage of 45-50% (Fig. S2A) in the three biological replicates. In these analyses, we found the Cdk1 site pS36 and pS11 (Fig. 2C), which we manually validated by reading the MS/MS fragmentation pattern (Fig. S2B).

### Spc110 phosphorylation at S11 and S36 are important for timely cell cycle progression

Spc110 phosphosite pS11 has been recently reported in a high-throughput study using DNA damage conditions (Lanz et al., 2021), but its function remains unknown. To investigate the biological functions of Spc110 pS11, we generated yeast mutants with the following non-phosphorylatable point mutations using CRISPR/Cas9: S11A, S36A, and S11AS36A. None of the mutants showed a growth defect at 16, 30 or 37°C (Fig. S3). Since *SPC110* is essential for growth, these results indicate that the mutants do not affect the protein's expression and folding.

To assess the effect of the *SPC110* mutant alleles in spindle formation and cell cycle progression, we imaged cells expressing Spc110-superfolderGFP (sfGFP) [wild-type (WT) and mutant alleles] and mRuby2-Tub1 (α-tubulin, MT marker). We synchronised the cells in G1 with α-factor, released them in fresh media, and fixed them at 10-min intervals for microscopy. Spc110-sfGFP had the same localisation and intensity in the mutants as the WT strain (Fig. S4), indicating that the mutations do not disrupt the expression and SPB localisation of the protein.

**Fig. 3.**
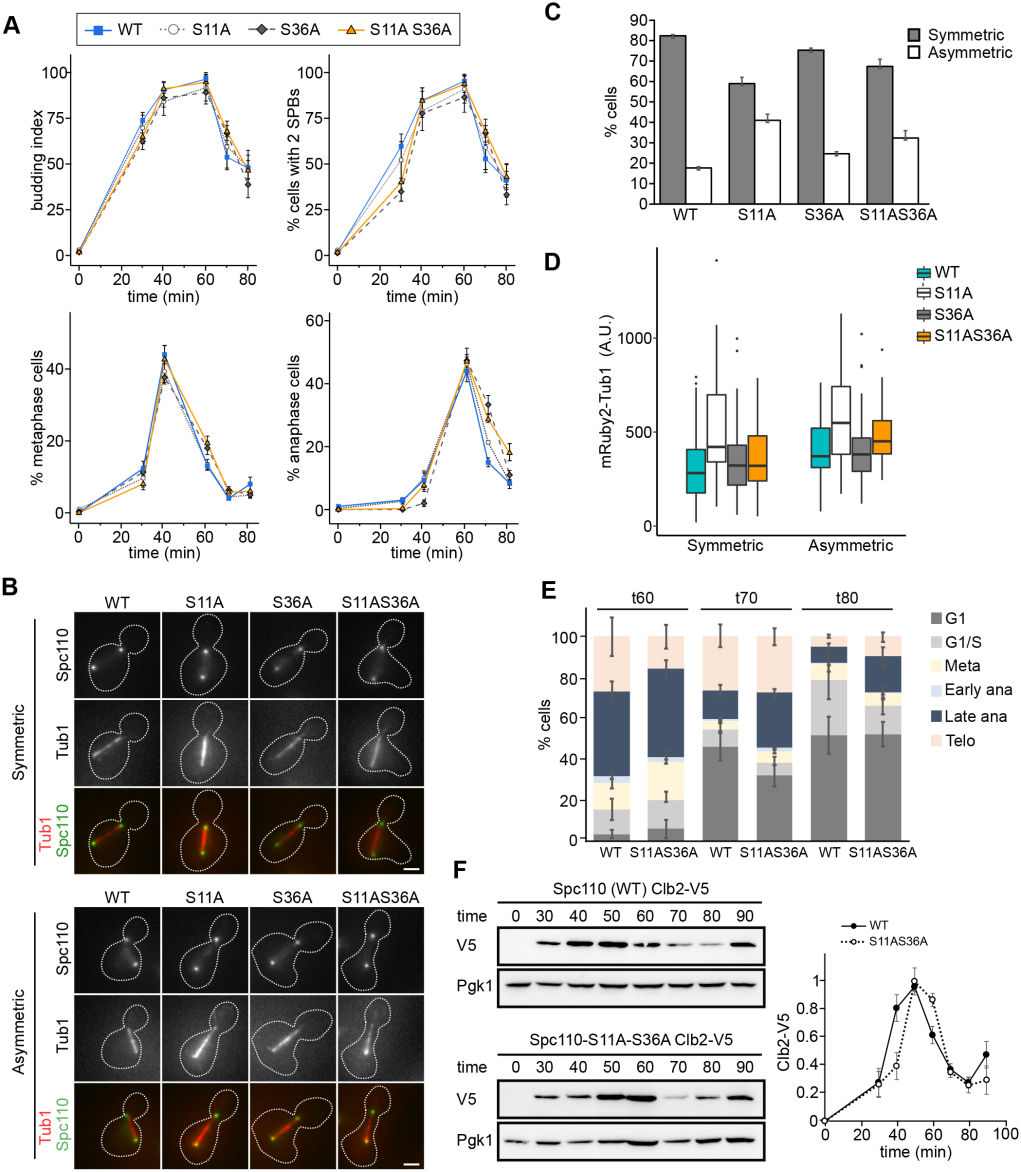
**Spc110^S11A S36A^ cells have a cell cycle delay.** (A) Spc110-sfGFP (WT) and derivative mutants (Spc110^S11A^-sfGFP, Spc110^S36A^-sfGFP, and Spc110^S11A S36A^-sfGFP) expressing mRuby2-Tub1 were arrested in G1 with α-factor, released in fresh media, and cells were fixed at different times after the release. The budding index (% of cells with a bud) and the number of cells with duplicated SPBs were measured. Cells were scored for cell cycle progression based on bud size and spindle length, and the percentage of cells in metaphase and anaphase are shown. Bars represent means, and error bars are ±s.e.m. (*n*=3 independent experiments, 100 cells each time point for each strain analysed per experiment). (B) Representative images of early anaphase cells (same strains as in A, 50 min post-release) displaying symmetric (upper panel) and asymmetric (lower panel) spindles. Images shown are maximum intensity projections of Z stacks. Scale bar: 2 µm. (C) Cells were treated as in B and the numbers of cells displaying each class of spindles (symmetric and asymmetric), based on the distribution of mRuby2-Tub1, was scored. Error bars are ±s.d. *n*=3 independent experiments, 100 cells of each strain analysed per experiment. Statistical significance for the frequency of asymmetric spindles was calculated by one-way ANOVA (*P*<0.001) with Tukey's multiple comparison *post hoc* test (κ=4, ν=8), and all pairs have *P*<0.01, except S11A versus S11AS36A, which has *P*<0.05. (D) mRuby2-Tub1 was quantified in the same images as in (B-C). Boxes correspond to the 25th and 75th percentile of values. The horizontal lines within the boxes represent the median. Whiskers extend to the largest/smallest value within 1.5* interquartile range. One of three independent experiments is shown. Number of cells: 100 cells per strain. Welch’s two-sample *t*-test (two-sided) was performed for the WT and S11A mutant, *P*-value= 4.924e-07 (symmetric spindles) and *P*-value=0.006929 (asymmetric spindles). (E) Stacked bar graphs showing the percentage of cells in each cell cycle phase for the same samples shown in A (60, 70 and 80 min after release). Bars are the mean, and error bars are ±s.d. (*n*=3 independent experiments, 100 cells per time point for each strain analysed per experiment). (F) Comparison of Clb2 levels in Spc110-sfGFP (WT) and Spc110^S11A S36A^ cells. Spc110 (WT) and Spc110^S11A S36A^ cells expressing mRuby2-Tub1 and Clb2-3xV5 were arrested in G1 with α-factor, released in fresh media, and whole-cell extracts were prepared at different times. Left: western blot analyses with anti-V5 and anti-Pgk1 (loading control) antibodies. One of three independent experiments is shown. Right: densitometry results. All bands were quantified and normalised with respect to their Pgk1 loading control and expressed relative to the maximal value within the time course (1). The values shown are mean±s.e.m. (*n*=3 independent experiments).

Next, we measured the cell cycle progression during the release from G1-arrest. The budding index (percentage of cells with a bud) plots were similar for WT and mutant cells, indicating that all the cells exit the G1 arrest with similar kinetics (Fig. 3A, first graph). The number of cells with duplicated SPBs showed a delay in SPB separation that was most evident for the S36A and S11AS36A strains (Fig. 3A, second graph, t30).

We then scored the number of cells in each cell cycle phase according to the spindle length (mRuby2-Tub1). Progression through metaphase was similar for all cells, with a sharp peak at 40 min post-release (Fig. 3A, third graph). Likewise, all the anaphase plots had a sharp peak 60 min post-release (Fig. 3A, fourth graph). By contrast, the anaphase peak was slightly broader in Spc110^S11AS36A^-sfGFP cells and had a higher number of cells in anaphase than WT cells at 70 min (29% versus 15%) and 80 min (18% versus 8%).

When scoring the cell cycle progression, we noticed that the Spc110^S11A^ spindles were more robust than the spindles of the other strains (Fig. S4). The difference was most noticeable in early anaphase and persisted in late anaphase. Therefore, we further examined the spindle morphology in early anaphase (Fig. 3B-D). We observed two different classes of spindles: symmetric (tubulin equally distributed between the two SPBs) and asymmetric (less tubulin at the spindle end closer to the bud) (Fig. 3B). Next, we quantified the frequency of symmetric and asymmetric spindles (Fig. 3C). In WT cells, most spindles were symmetric, and a few cells (14%) had asymmetric spindles. The frequency of asymmetric spindles increased severely (44%) in the S11A (23%) mutant, and to a lesser extent in the S11AS36A (30%) and S36A (23%) mutants. We then measured the amount of tubulin based on the fluorescence intensity of mRuby2-Tub1 at the spindle (Fig. 3D). The spindles in the S11A mutant had a higher tubulin intensity than the rest of the strains, both in symmetric and asymmetric spindles (Fig. 3D).

To analyse in more detail the cell cycle delay of the S11AS36A mutant, we plotted the distribution of all cell cycle stages at 60-, 70- and 80-min post-release (Fig. 3E). In WT cells, at 60 min, most cells had a late anaphase phenotype, with elongated spindles and SPBs segregated between mother and bud. The percentage of late anaphase cells rapidly decreased over time, leading to a higher percentage of G1 and G1/S cells at 70 and 80 min. Comparing Spc110^S11AS36A^-sfGFP with WT cells, the S11AS36A mutant has an increase in late-anaphase at t70 and t80, indicating a slower cell cycle progression.

Finally, we explored the cell cycle progression of WT and mutant cells by following the expression of the mitotic cyclin Clb2 during the release (Fig. 3F). Clb2 is expressed during mitosis and has different roles, such as activating APC/C-Cdc20 and elongating the mitotic spindle (Rahal and Amon, 2008), and it is depleted at the end of mitosis (Bäumer et al., 2000; Irniger et al., 1995; Schwab et al., 1997). Both the accumulation and degradation of Clb2 took place slightly later in the Spc110^S11AS36A^ than in WT cells, which is consistent with the cell cycle delay we observed in the mutant cells (Fig. 3A,E).

### Spc110 phosphorylation at S11 and S36 are not required for SPB inheritance

**Fig. 4.**
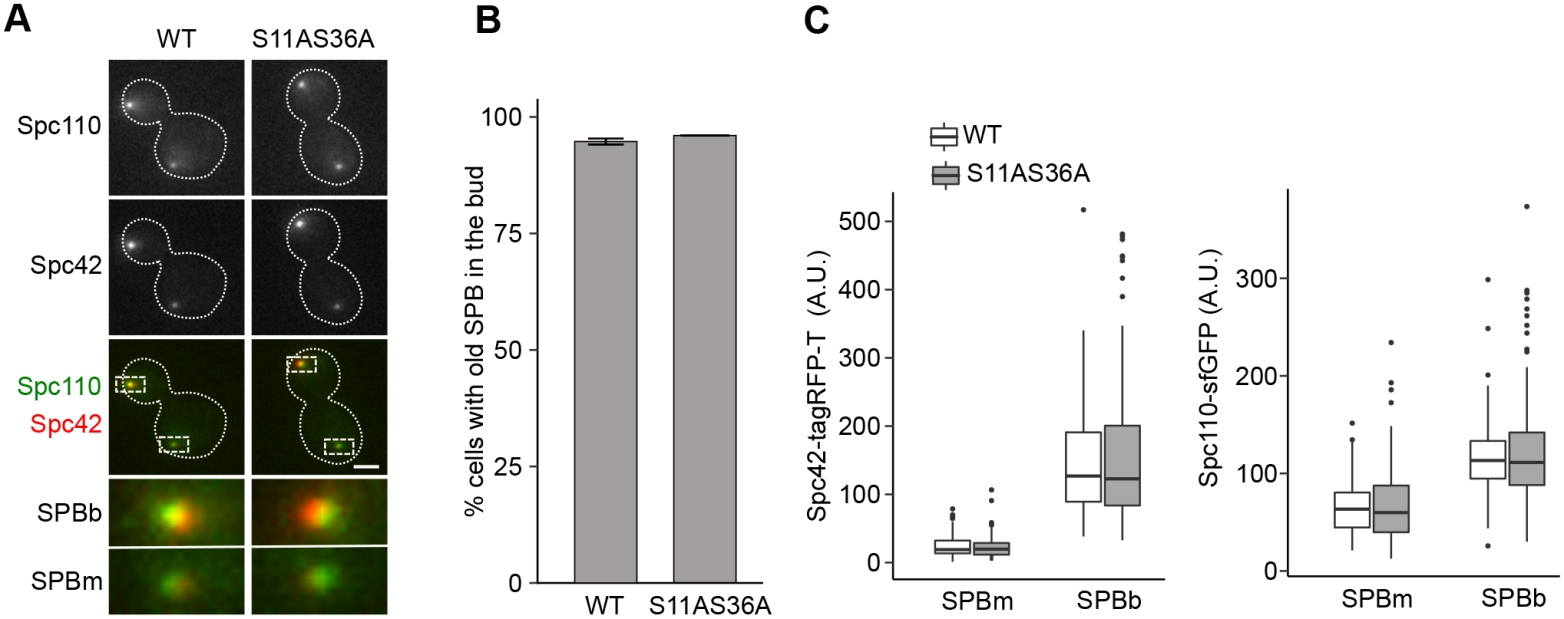
**Spc110^S11AS36A^ cells have the canonical SPB inheritance pattern.** (A) Representative images of Spc110-sfGFP-WT and Spc110**^S11A S36A^**-sfGFP cells expressing Spc42-tagRFP-T, exhibiting wild-type SPB distribution pattern (old SPB segregated to bud cells). Images shown are maximum intensity projections of Z stacks. Dashed boxes show area around the SPBs enlarged in the images below. Scale bar: 2 µm. (B) The percentage of cells displaying canonical SPB inheritance, based on Spc42-tagRFP-T distribution. Bars represent means, and error bars are ±s.d., *n*=3 independent experiments, 100 cells per time point for each strain analysed per experiment. (C) Quantification of Spc42-tagRFP-T and Spc110-sfGFP at SPBs. The amount of Spc42 and Spc110 was measured from maximum intensity projections in late anaphase cells. Boxes correspond to the 25th and 75th percentile of values. The horizontal lines within the boxes represent the median. Whiskers extend to the largest/smallest value within 1.5* interquartile range. One of three independent experiments is shown. Number of cells: 100 cells per strain.

Next, we investigated the inheritance of SPBs in the Spc110^S11AS36A^ strain. The age-dependent SPB inheritance is determined by the SPB outer plaque components Nud1 and Spc72 (Hotz et al., 2012; Lengefeld et al., 2017; Manzano-López et al., 2019; Matellán et al., 2020). Yet, inner plaque SPB components could also have an indirect effect. To measure the SPB inheritance, we co-expressed Spc110-sfGFP (WT and S11AS36A) with Spc42-tagRFP-T (Fig. 4A). The slow maturation of RFP makes the old SPB appear brighter than the newly synthesised one (Pereira et al., 2000). We scored the number of late anaphase cells with the brightest Spc42 (old SPB) in the bud and showed that Spc110^S11AS36A^ displayed normal inheritance (Fig. 4B). Furthermore, we quantified the fluorescence intensity of Spc42-tagRFP-T and Spc110-sfGFP at SPBs in mother and bud cells and found comparable levels in WT and S11AS36A mutant cells (Fig. 4C). Thus, Spc110^S11AS36A^ proteins are segregated like Spc110-WT without affecting the SPB inheritance.

### Spc110^S11AS36A^ cells have a delayed mitotic exit

Our microscopy analyses showed that Spc110^S11AS36A^ cells have normal segregation and inheritance of SPBs but a slower progression through the cell cycle. We next investigated the localisation of the phosphatase Cdc14, which in budding yeast has a key role in promoting mitotic exit by counteracting the activity of Cdk1.

**Fig. 5.**
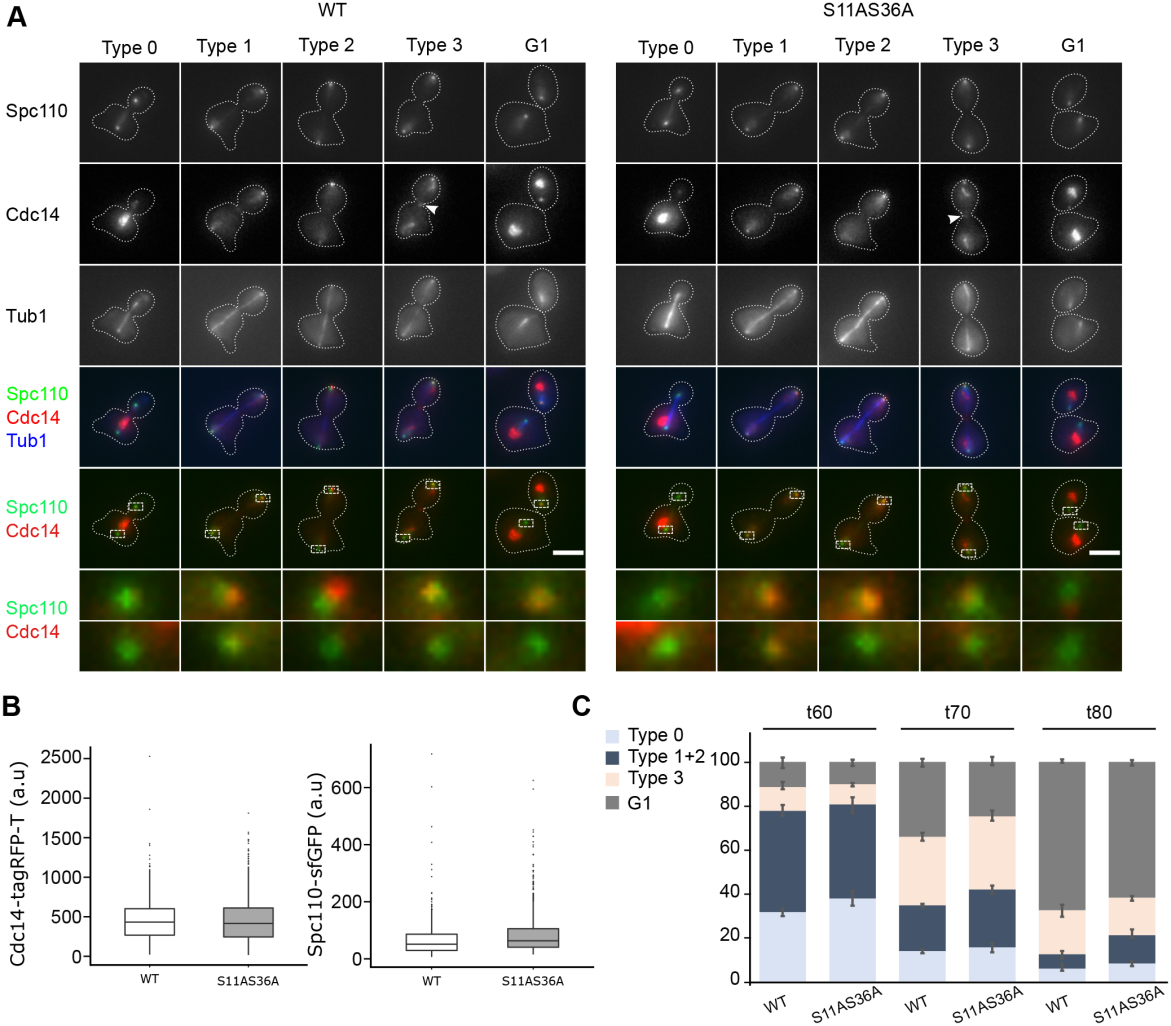
**Cdc14 localisation shows a delayed mitotic exit in Spc110^S11A S36A^ cells.** (A-C) Spc110-sfGFP and Spc110^S11A S36A^-sfGFP cells expressing Cdc14-tagRFP-T and mTurquoise2-Tub1 were arrested in G1 with α-factor, released, and fixed after 0, 60, 70 and 80 min. (A) Representative images of Cdc14 localisation patterns in late anaphase-G1 cells: nucleolar (Type 0), SPBs (Type 1), bud SPB (Type 2), bud SPB, nucleoli and bud neck (Type 3) and nucleolar and bud SPB (G1). Images are maximum intensity projections of Z stacks. Dashed boxes indicate the area around the SPBs enlarged in the images below. Scale bars: 2 µm. (B) Boxplots of the integrated fluorescence intensity of Cdc14-tagRFP-T (left) and Spc110-sfGFP (right) at SPBs in G1-arrested cells. Boxes correspond to the 25th and 75th percentile of values. The horizontal lines within the boxes represent the median. Whiskers extend to the largest/smallest value within 1.5* interquartile range. Number of cells: WT-Cdc14 (726), S11AS36-Cdc14 (1004), WT-Spc110 (670), and S11AS36A-Spc110 (806). (C) Stacked bar graphs showing the percentage of cells with each Cdc14 localisation pattern in late anaphase-G1 cells. Bars are the mean and error bars are ±s.e.m. (*n*=3 independent experiments, 200 cells per time point for each strain).

We co-expressed Cdc14-tagRFP-T in Spc110-sfGFP (WT) or Spc110^S11AS36A^-sfGFP cells expressing the tubulin marker mTurquoise2-Tub1. We arrested the cells in G1 with α-factor, released the cells, and followed the localisation of Cdc14 from 60-80-min post-release when most cells are in late anaphase (Fig. 5A-C). We verified that WT and Spc110^S11AS36A^ cells had comparable levels of Cdc14 and Spc110 in G1 arrested cells, where Cdc14 is sequestered in the nucleoli and cells have one SPB (Fig. 5B).

During the release, we observed different patterns of Cdc14 localisation (Fig. 5A). In late anaphase cells with shorter spindles, Cdc14 was at the nucleoli (Type 0). By contrast, in late-anaphase cells with long spindles, where the bud SPB had reached the bud cortex, Cdc14 was predominantly found at both SPBs (Type 1) or at the bud SPB (Type 2). In telophase cells characterised by disassembled spindles, Cdc14 was localised at the bud SPB, nucleoli and bud neck (Type 3). Finally, after cytokinesis, Cdc14 re-localised to the nucleoli and was partly retained at SPB in the bud (G1). Thus, since Cdc14 localisation at the SPBs requires the activation of the MEN pathway (Yoshida et al., 2002) we measured the percentage of WT and mutant cells with the following Cdc14 localisations: Type 0 (before MEN activation); Types 1-2 (after MEN activation); Type 3 (telophase cells, before cytokinesis) and G1 (after mitotic exit) (Fig. 5C). As expected, late anaphase cells in the WT strain progressed through the different Cdc14 localisation types during the release, starting with 31% Type 0 cells at 60 min and reaching 68% G1 cells at 80 min. Spc110^S11AS36A^ cells followed the same pattern but were slightly delayed, starting with 38% Type 0 cells and reaching 60% cells in G1.

Lastly, we repeated the cell cycle and Cdc14 analyses in α-factor synchronised cells by adding α-factor during the release once the cells had budded (40 min post-release), thereby arresting the cells in the next G1 (Fig. S5A-B). This experimental set-up allowed us to look at one synchronous cell cycle. We found that Spc110^S11AS36A^ cells have a slower cell cycle progression resulting in a delay entering the next G1 (Fig. S5A), in agreement with our previous results (Fig. 3). The delay was most evident 70 min post-release when the percentage of cells in G1 was 50% in WT cells versus 32% in Spc110^S11AS36A^. Likewise, scoring the localisation of Cdc14-tagRFP-T confirmed that Spc110^S11AS36A^ have a delayed mitotic exit (Fig. S5B). Specifically, 70 min post-release, WT had a predominant Cdc14-G1-localisation (61%) and a small percentage of Type3 cells (6%), while Spc110^S11AS36A^ had fewer cells with Cdc14-G1 localisation (36%) and a higher percentage of Type 3 cells (30%).

## DISCUSSION

In this work, we applied the RITE method to separate old (Flag-tagged) and new (V5-tagged) Spc110 with four steps: (1) RITE-assay, (2) TAP-enrichment of intact SPBs, (3) denaturing SPB complex and (4) Flag- and V5-immunoprecipitation. Further mass spectrometry analyses identified three phosphosites (S11, S36, and S60) in Spc110-Flag. Therefore, considering the flow cytometry analysis of our samples (Fig. 1C), we interpret that S11, the Cdk1-dependent phosphosite S36 and the Mps1-dependent phosphosite S60 are phosphorylated in old (from maternal origin) Spc110 at G2/M. The low sequence coverage within the region where other activating sites lie (Fig. S2A) prevented us from identifying the rest of the previously described activating phosphosites (pT64, pT68 and pS91).

The S11 phosphosite of Spc110 was absent in previous comprehensive phospho-proteomic analyses of the SPB (Fong et al., 2018; Keck et al., 2011). One possible explanation for this is that in those studies, cells were treated with nocodazole (Keck et al., 2011) and Cdc20 depletion (Fong et al., 2018) and thereby arrested at the metaphase–anaphase transition. By contrast, cells are only partly synchronised (80% in G2/M) and not arrested with our nutrient-depletion strategy. Thus, if S11 phosphorylation is a transient modification that occurs in anaphase, our approach would be more likely to detect it.

Cdk1 phosphorylation at pS36 (together with pS91) occurs from S-phase and stimulates γ-TuSC nucleation and spindle assembly (Lin et al., 2014). Following this role, the non-phosphorylatable mutants Spc110^S36A^ and Spc110^S11AS36A^ had a delayed SPB separation (Fig. 3A, second graph, t30). Furthermore, anaphase was lengthier in Spc110^S36A^ and Spc110^S11AS36A^ mutants (Fig. 3A, fourth graph). By contrast, the non-phosphorylatable mutant Spc110^S11A^ had a normal cell cycle progression. Yet, we found that Spc110^S11A^ cells have a distinct phenotype characterised by spindles that are more robust than WT spindles and frequently asymmetric, with less tubulin near the old SPB (Fig. 3B-C). Overall, these data suggest that pS11 and pS36 have different physiological functions, where pS36 has a critical role in spindle assembly and elongation, while pS11 might be involved in maintaining the symmetry of the spindle and regulating tubulin turnover.

The predominant phenotype in the double mutant Spc110^S11AS36A^ was a delay in late anaphase and telophase (Fig. 3B; Fig. S5A), indicating a dominant effect of the pS36A mutation. Furthermore, following Cdc14 localisation, we measured that Spc110^S11AS36A^ had a slower Cdc14 exit from the nucleolus (Fig. 5E, t60, higher percentage of Type 0 cells) and re-entry in G1 (Fig. S5B, t70). The N-terminal domain of Spc110 anchors the γ-tubulin complex to the nuclear side of the SPB (Knop and Schiebel, 1997) and is required for oligomerization of budding yeast γ-TuSC *in vitro* (Kollman et al., 2010). A possible explanation for the observed delay in Spc110^S11AS36A^ is a failure in modulating microtubule dynamics or γ-TuSC affinity during late anaphase, resulting in a slower elongation of the mitotic spindle, and a delay in MEN activation and Cdc14 release from the nucleolus. Indeed, the Spc110^S36A^ mutant was reported to affect spindle dynamics in anaphase (Huisman et al., 2007).

Interestingly, we searched for kinase consensus sites in Spc110 using Scansite 4 (https://scansite4.mit.edu) and the protein kinase Cdc15, the effector of the MEN pathway, was predicted as a potential kinase for Spc110-S11. Therefore, we hypothesise that phosphorylation of Spc110 at S11 is a target of the MEN pathway. This would explain why we identify this phosphosite in old (Flag-tagged) Spc110, primarily located at the old SPB where MEN is activated. However, during MEN activation, Cdc15 is docked to the cytoplasmic side of the SPB. It remains to be investigated whether there is an active pool of Cdc15 in the nucleus.

Spc110 is a conserved protein structural related to the centrosomal proteins pericentrin and centrosomin (Lin et al., 2014). Therefore, future studies to uncover the regulation of Spc110 phosphosites will shed light on our general understanding of mitosis.

## MATERIALS AND METHODS

### Yeast strains and plasmids

All the *S. cerevisiae* strains in this study are derivatives of S288C (Table S1). Plasmids and primers are listed in Tables S2 and S3. Genes (*SPC110*, *SPC97*, *SPC42*, *CDC14* and *CLB2*) were tagged by PCR-mediated homologous recombination and confirmed by sequencing. The mRuby2-Tub1 and mTurquoise2-Tub1 strains were constructed by integrating the plasmids pHIS3p-mRuby2-Tub1 (Addgene #50645) and pHIS3-pmTurquoise-Tub1 (Addgene #50641) at the TUB1 locus, as previously described (Markus et al., 2015).

The mutations in SPC110 were introduced using CRISPR/Cas9 (Levi and Arava, 2020). For each PAM target site, two complementary 25-bases long oligonucleotides were designed by ATUM gRNA Design Tool, synthesised with flanking BplI sites, and cloned into the plasmid bRA66 (Addgene #100952) using BplI (Thermo Fisher Scientific, ER1311) and T4 ligase (New England BioLabs, M0202S). Yeasts were co-transformed with the gRNA-bRA66 constructs and 80 bp oligonucleotides (donor DNA) using LiAc (32). To allow for loss of the bRA66-gRNA plasmid, overnight cultures were diluted to OD_600_= 0.1, grew for 6 h at 30°C and hygromycin sensitive colonies were selected and sequenced.

### Growth conditions

Cells were grown at 30°C in YEP (1% yeast extract, 2% bactopeptone) medium supplemented with 2% glucose. For synchronisation, overnight cultures were diluted to OD_600=_0.1, grown for 3 h and arrested by adding 3 µg/ml α-factor (Sigma, custom peptide WHWLQLKPGQPMY) twice, with a 60 min interval. Cells were released by washing with YEP, resuspending in YEP, and incubating at 30°C. To rearrest the cells in the next G1, 3 µg/ml α-factor was added again to the cultures 40 min after the release.

### Flow cytometry

Cells (1 ml) were fixed in 70% ethanol on ice for 1 h, resuspended in 50 mM sodium citrate (pH 7) for 15 min at RT, and incubated with 10 mg/ml RNase A (Sigma, 10109134001) overnight at 37°C. The cells were washed with PBS, stained with 50 µg/ml propidium iodide (PI, Sigma 11348639001) in 100 µl PBS, and sonicated for five pulses (2 s on, 2 s off, 25% power). DNA content was analysed using a flow cytometer (Cytoflex, Beckman Coulter) and FlowJo software.

### RITE-based affinity purification of old and new Spc110

The RITE-genetic switch was induced as described before (Menendez-Benito et al., 2013). Briefly, cells were grown in 500 ml of YEP supplemented with 2% glucose and hygromycin (200 µg/ml) for 48 h at 30°C, spun down and resuspended in 1×volume of YEP (without glucose) with 1 µM β-estradiol (1914275, Sigma-Aldrich) to induce the genetic switch overnight. The cells were then released in 10 L of YEP media with 2% glucose and grown at 30°C.

For protein purification, the cells were pelleted, resuspended in 800 μl TAP purification buffer (TAP: 50 mM Tris, 150 mM NaCl, 5% Glycerol and 0.1% TritonX) and lysed in a SPEX^TM^ SamplePrep Freezer/Mill, using 10 rounds of 2 min grinding at 14 counts per second (cps) and 2 min cooling. Spc97-TAP purification of SPBs was performed as previously described (24) and the SPBs were eluted with 5 μg proTEV plus protease (Promega, V6101) in TEV buffer overnight at 4°C. Trichloroacetic acid (TCA) was added to a 10% concentration, proteins were precipitated on ice for 1 h and washed with ice-cold acetone. The pellet was air-dried, dissolved in B0 buffer (100 mM Tris-HCl, 2% SDS, 0.05% bromophenol blue, 5% glycerol), and boiled at 95°C for 10 min.

For affinity purification, the samples were diluted in IP buffer (50 mM sodium HEPES, 300 mM NaCl, 55 glycerol, 0.5% triton-100, 1 mM MgCl2, 1 mM DTT, 1 mM PMSF, 1 mM leupeptin, 1 mM pepstatin A, 10 mM NaF, 1 mM sodium pyrophosphatase, 1 mm sodium orthovanadate), and incubated with Protein G Dynabeads (Thermo Fisher Scientific) conjugated with Flag M2 mouse monoclonal antibody (Sigma, F1804, 1:5000) or V5 tag mouse monoclonal antibody (Thermo Fisher Scientific, R960-25, 1:5000) at RT for 30 min. The beads were washed with wash buffer (50 mM Tris-HCl, 1 mM EDTA, 150 mM NaCl and protease inhibitors) four times.

### Western blot analysis

Proteins were separated by SDS-PAGE, transferred to a PVDF membrane (BioRad) and immunoblotted with primary monoclonal anti-FLAG M2 antibody (Sigma-Aldrich, F3165, diluted 1:5000); V5 tag mouse monoclonal antibody (Thermo Fisher Scientific, R960-25, diluted 1:5000), Pgk1 monoclonal mouse antibody (Invitrogen, 22C5D8, diluted 1:10,000) and HRP-conjugated secondary antibodies, detected with Femto maximum sensitivity substrate (Thermo Fisher Scientific). ImageLab software (Bio-Rad) was used for image acquisition and densitometric analysis. The density of a given band was measured as the total volume under the peak and corrected with background subtraction. The intensity was normalised with respect to corresponding Pgk1 signals of each sample.

### Sample preparation for mass spectrometry analysis

Samples were denatured and reduced with 50% trifluoroethanol (TFE) and 20 mM Tris (2-carboxyethyl) phosphine (TCEP) in 50 mM triethylammonium bicarbonate (TEAB) for 20 min at 55°C, then alkylated by 55 mM chloroacetamide (CAA) at RT for 30 min in the dark, followed by digestion with 5 mg of Lys C for 4 h and 5 mg modified trypsin overnight at 37°C. After digestion, the samples were centrifuged at 20,000 g for 10 mins to collect the peptide supernatant. The peptides were acidified with 10% trifluoroacetic acid (TFA) to pH 2, then desalted with a C18 StageTip (Empore 3 M, 2215). The stage tip was activated with 100% acetonitrile and equilibrated with 0.5% acetic acid. The peptide sample was then loaded onto the column and washed with 0.5% acetic acid. The bound peptides were eluted with 65% acetonitrile in 0.5% acetic acid solution into a fresh 1.5 ml Eppendorf tube.

Phosphopeptides were enriched from the desalted peptides with TiO_2_ beads. The samples for phospho-enrichment were resuspended to the final composition of 6% TFA in 40% acetonitrile and incubated with the beads at room temperature (RT) for 10 mins. This incubation was repeated once with fresh beads. The beads were loaded over a C8 mesh, washed with 6% TFA in 10%, 40% and 60% acetonitrile sequentially, and eluted twice with 5% ammonia water first, followed by 5% ammonia water in 25% acetonitrile. The phosphopeptides were acidified and filtered to remove the TiO_2_ beads with a C8 frit prior to sample acquisition.

### Mass spectrometry

Non-enriched total peptides were analysed by mass spectrometry analysis using reverse-phase liquid chromatography on a Proxeon 1000 UHPLC system connected to Orbitrap Fusion Lumos mass spectrometer (Thermo Fisher Scientific). Each fraction was separated on 50 cm×75 µm Easy Spray column (Thermo Fisher Scientific) in a 70-min gradient with a pre-programmed mixing of solvent A (0.1% formic acid in water) and solvent B (99% acetonitrile, 0.1% formic acid in water). The following acquisition parameters were applied: data-dependent acquisition in positive mode with survey scan of 120,000, scan range of 350-1550 m/z, and AGC target of 4e5; MS/MS collision-induced dissociation in an ion trap and AGC target of 5e4; isolation window 1 m/z.

All phosphopeptides samples were subjected to mass spectrometry analysis using reverse-phase liquid chromatography on an nLC 1000 UHPLC system connected to Q Exactive HF-X Quadrupole-Orbitrap mass spectrometer (Thermo Fisher Scientific). Separation column and solvent gradient setting are the same as above-mentioned on Lumos mass spectrometry. The only different acquisition parameters were: AGC target of 5e5 for full MS scan; and 32% HCD energy used for precursor fragmentation.

### Mass spectrometry data analysis

Peak lists were generated in Proteome Discoverer 2.3 software (Thermo Fisher Scientific) with Mascot 2.6.0 (Matrix Science) and concatenated forward/decoy *Saccharomyces cerevisiae* database (7939 entries). Search parameters: MS precursor mass tolerance 10 ppm, MS/MS 0.06 Da, maximal three missed cleavages; static modifications: carbamidomethyl (C); variable modifications: oxidation (M), deamidated (NQ), phospho (ST), phospho (Y) and acetyl N-terminal protein. False discovery rate estimation with two levels: strict=1% FDR and medium=5% FDR.

### Fluorescence imaging

Cells were fixed in 4% formaldehyde at RT for 15 min, washed with PBS, and stored in PBS at 4°C. Fixed cells were then plated in concanavalin-A-coated 96-well glass-bottomed plates (Mo Bi Tec 5242-20) and imaged in PBS. Z-stacks (4.5 µm, 0.3 µm) were acquired using a Nikon Ti2 inverted microscope equipped with a Kinetix sCMOS camera and a 60× water immersion objective (1.20 NA).

### Image analysis

Maximum intensity projections of image z-stacks were made with FiJi. Cell cycle analyses were performed by classifying the cells: G0/G1 (unbudded); G1/S (budded, spindles <1 µm); metaphase (budded, spindles <1-2 µm); early anaphase (budded, spindles >2 µm; SPBs in mother cell); late anaphase (budded, spindles >2 µm, SPBs segregated); and telophase (budded, elongated, or disassembled spindles, SPBs segregated). The fluorescence intensities of Spc42-tagRFP-T and Spc110-sfGFP were quantified with FiJi, segmenting SPBs by Spc42-tagRFP-T intensity. The regions of interest (ROIs) were used to measure integrated fluorescence intensities in the red (Spc42-tagRFPT-T) and green (Spc110-sfGFP) channels. Background correction was calculated by multiplying the mean fluorescence in the cytosol with the ROI's area and subtracting it from the integrated intensity. Cdc14-tagRFP-T, Spc110-sfGFP and mRuby2-Tub1 fluorescence intensities were quantified using the same approach, segmenting areas of G1 SPBs (using Spc110-sfGFP signal) or G1 nucleoli (using Cdc14-tagRFP-T signal). Early anaphase spindles were analysed by first applying a directional filter and then segmenting using the mRuby2-Tub1 signal intensity.

## Supplementary Material

10.1242/biolopen.059565_sup1 Supplementary information
