# Supplementary material for: Phosphosites of the yeast centrosome component Spc110 contribute to cell cycle progression and mitotic exit
Source: Biol Open. 2022 Nov 7;11(11):bio059565. doi: 10.1242/bio.059565 (PMC9672857; doi:10.1242/bio.059565)
Supplement: Supplementary information [file biolopen-11-059565-s1.pdf]

Fig. S1.

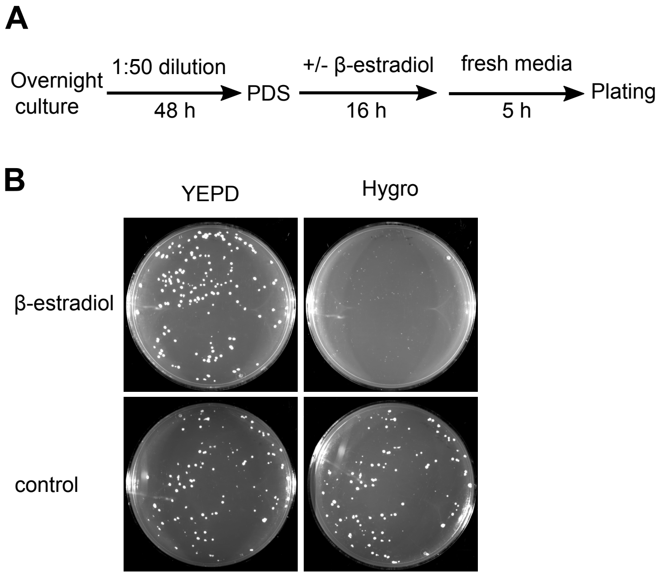

Fig. S1. Spc110-RITE plating assays and protein purification in native conditions. (A) Plating assay to assess the efficiency and spontaneous recombination of the RITE assay as shown in Fig. 1B-C. Briefly, cells were grown to the PDS stage, the culture was split into two, and the genetic switch was induced in one of the cultures by adding β-estradiol to the media, while leaving the other culture untreated (+/- β-estradiol). Then, the cells were released in fresh media for 5 hours and cells were plated without selection on YEPD plates, grown, and replicated onto control plates (YEPD) and plates with hygromycin (HYGRO). (B) Representative plates of the plating assay. The percentage of colonies with hygromycin sensitivity in the cultures with β-estradiol (i.e., switch efficiency) was 100%(top panel), while percentage of cells that become hygromycin resistant in the absence of β-estradiol (spontaneous recombination) was 8% (bottom panel). One representative experiment of three independent experiment is shown.

Biology Open • Supplementary information

Fig. S2.

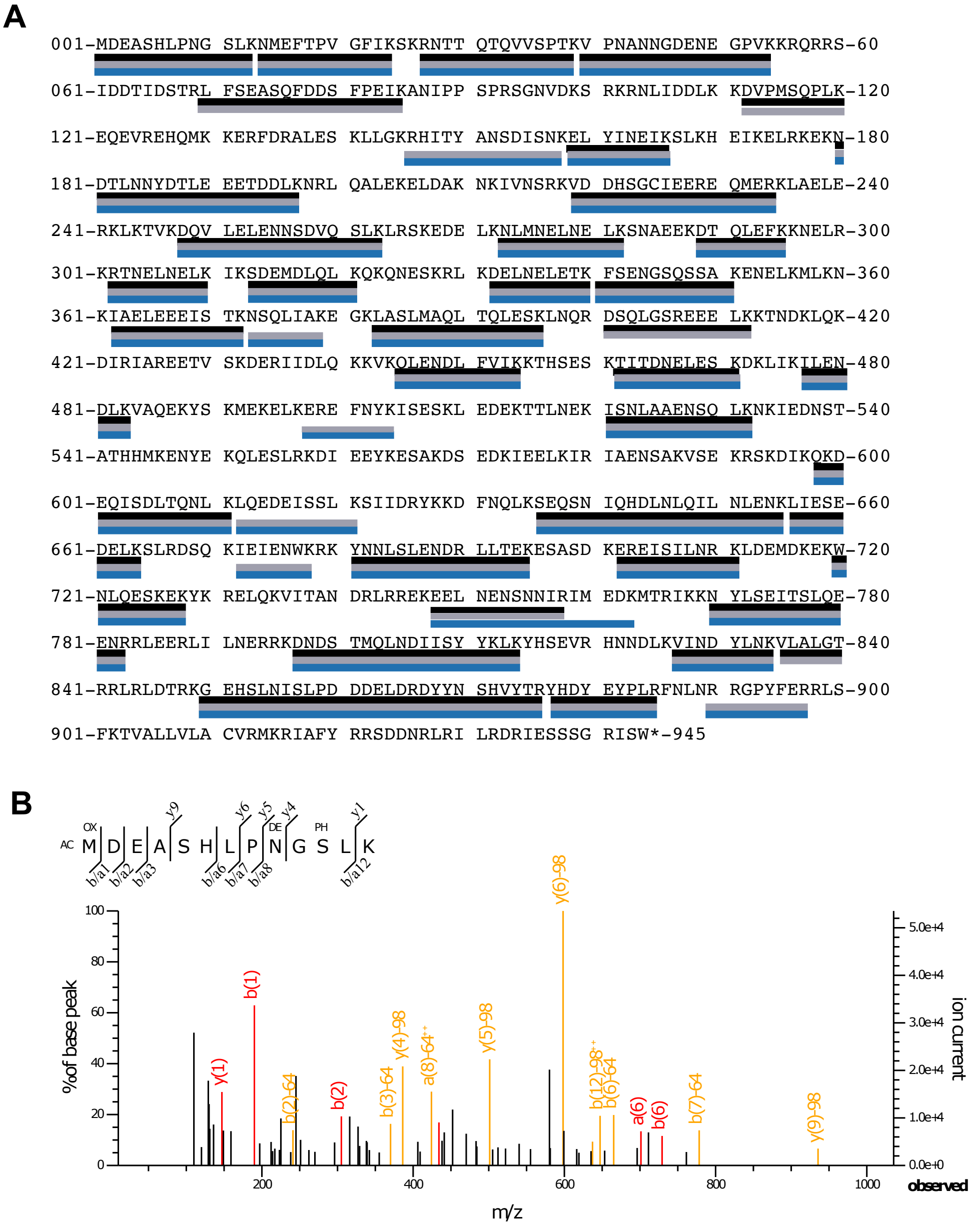

Fig. S2. Mass spectrometry of Spc110-Flag purified samples. (A) Spc110 sequence coverage by mass spectrometry of the Spc110-Flag purified sample. The coverage from each replicate is indicated by different colors (B) Phosphorylation on a serine residue Ser11 in the SPC110 peptide with the highest sequence-fitting score (36) revealed by MS/MS of 2+ molecular ions (M). y9, y6, y5 and y4 are shown as fragments carrying the modification.

Biology Open • Supplementary information

Fig. S3.

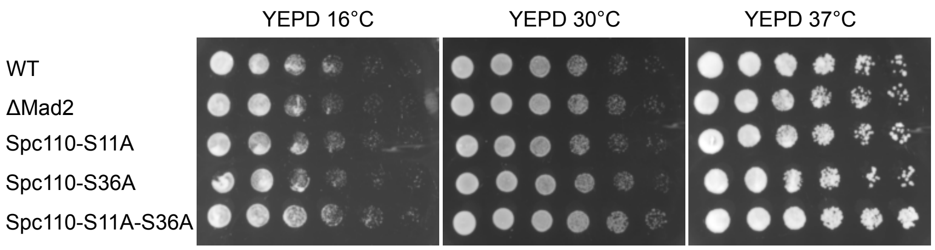

Fig. S3. Spotting assays of Spc110 phosphomutant yeasts. Spotting test assays show equal growth and sensitivity to heat shock for Spc110-WT and the mutant derivatives. Serial 5-fold dilutions of isogenic wild type cells, single-, double-mutants and Δmad2 were spotted on YEPD plates and grew at different temperatures. WT, wild-type strain.

Biology Open • Supplementary information

Fig. S4.

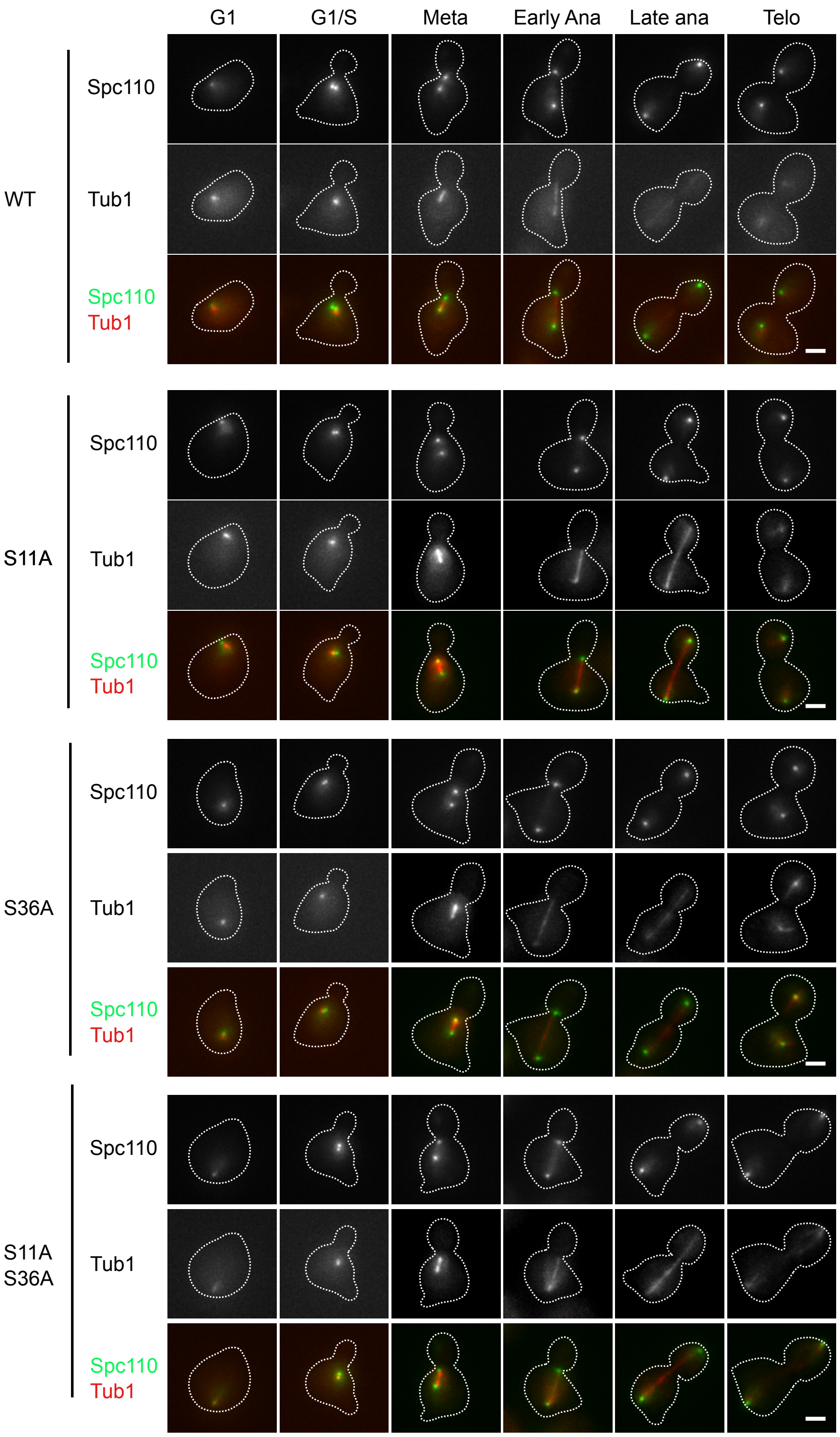

Fig. S4. Spc110 and Tub1 have the same distribution through the cell cycle in Spc110-WT and mutant strains. Representative images of Spc110-sfGFP (WT) and derivative mutants (Spc110S11A-sfGFP, Spc110S36A-sfGFP, and Spc110S11A S36A-sfGFP) expressing mRuby2-Tub1. Cells were arrested in G1 with α-factor and released in fresh media, as indicated in Fig. 3A. Images shown are maximum intensity projections of Z stacks. Dashed lines represent the cell outlines based on bright field images. All scale bars 2 µm.

Fig. S5.

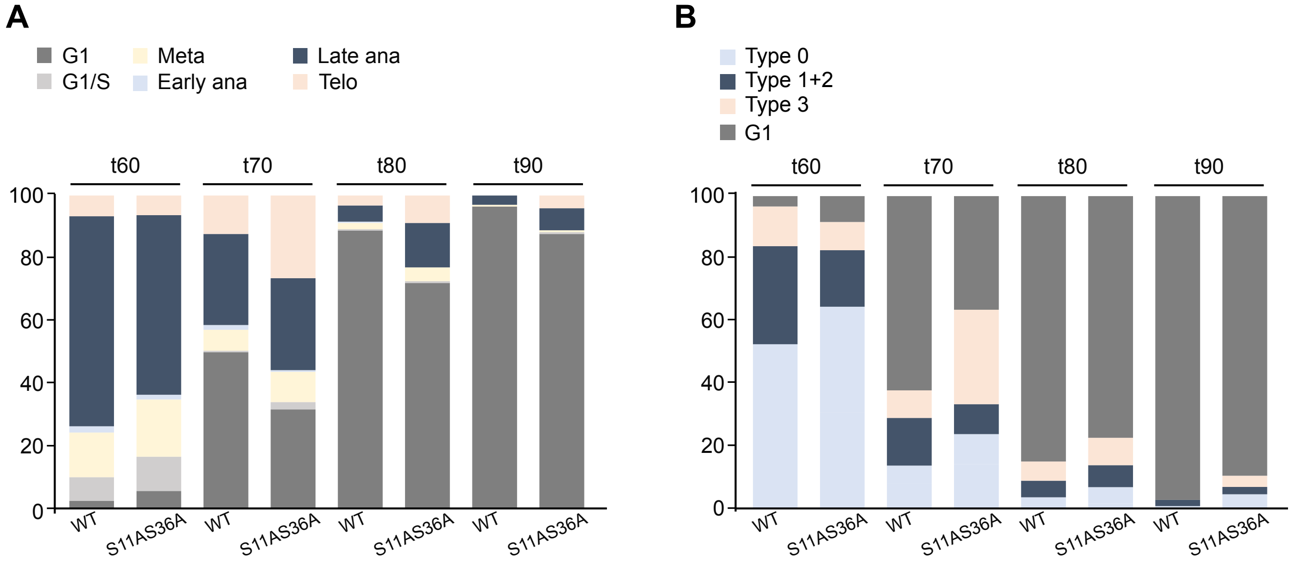

Fig. S5. Cdc14 localization shows a delayed mitotic exit in Spc110 S11A S36A cells. Spc110-sfGFP and Spc110S11A S36A-sfGFP cells expressing Cdc14-TagRFP-T and mTurquoise2-Tub1 were arrested in G1 with α-factor, released and, 40 min after the release, α-factor was added to the culture to arrest the cells in the next G1.(A) Stacked bar graphs showing the mean percentage of cells in each cell cycle phase (200 cells each time point for each strain). (B) Stacked bar graphs showing the mean percentage of cells with each Cdc14 localization pattern in late anaphase-G1 cells (200 cells each time point for each strain).

Table S1. Strains used in this study

Strain	Genotype
BY4141	MATa his3Δ1 leu2Δ0 lys2Δ0 ura3Δ0
NKI-5504	MAT@ his3Δ1 leu2Δ0 ura3Δ0 met15Δ0 can1Δ::STE2pr-Sp_his5 lyp1Δ::NATMX_TDH3pr_Cre-EBD78_CYC1t
VMB-097	MATa his3Δ1 leu2Δ0 ura3Δ0 met15Δ0 can1Δ::STE2pr-Sp_his5 lyp1Δ::NATMX_TDH3pr_Cre-EBD78_CYC1t, Spc110::SPC110-S-LoxP-5xFlag-HYG-LoxP-V5-ADH1t Spc97::SPC97-TAP-KIURA3
VMB-518	MATa his3Δ1 leu2Δ0 lys2Δ0 ura3Δ0 Spc110::SPC110-sfGFP-CaUra3
VMB-530	MATa his3Δ1 leu2Δ0 lys2Δ0 ura3Δ0 Spc110::SPC110-S11A-sfGFP-CaUra3
VMB-535	MATa his3Δ1 leu2Δ0 lys2Δ0 ura3Δ0 Spc110::SPC110-S11A-sfGFP-CaUra3 TUB1+3'UTR::HIS3p:mRuby2-TUB1-LEU2
VMB-538	MATa his3Δ1 leu2Δ0 lys2Δ0 ura3Δ0 Spc110::SPC110-sfGFP-CaUra3 TUB1+3'UTR::HIS3p:mRuby2-TUB1-LEU2
VMB-548	MATa his3Δ1 leu2Δ0 lys2Δ0 ura3Δ0 Spc110::SPC110-sfGFP-CaUra3 mad2Δ::HygroMX
VMB-565	MATa his3Δ1 leu2Δ0 lys2Δ0 ura3Δ0 Spc110::SPC110-S36A-sfGFP-CaUra3 TUB1+3'UTR::HIS3p:mRuby2-TUB1-LEU2
VMB-569	MATa his3Δ1 leu2Δ0 lys2Δ0 ura3Δ0 Spc110::SPC110-S11A-S36A-sfGFP-CaUra3
VMB-591	MATa his3Δ1 leu2Δ0 lys2Δ0 ura3Δ0 Spc110::SPC110-sfGFP-CaUra3 Cdc14::Cdc14-tagRFPT-KanMx
VMB-593	MATa his3Δ1 leu2Δ0 lys2Δ0 ura3Δ0 Spc110::SPC110-S11A-S36A-sfGFP-CaUra3 Cdc14::Cdc14-tagRFPT-KanMx
VMB-598	MATa his3Δ1 leu2Δ0 lys2Δ0 ura3Δ0 Spc110::SPC110-S11A-S36A-sfGFP-CaUra3 TUB1+3'UTR::HIS3p:mRuby2-TUB1-LEU2
VMB-617	MATa his3Δ1 leu2Δ0 lys2Δ0 ura3Δ0 Spc110::SPC110-sfGFP-CaUra3 Cdc14::Cdc14-tagRFPT-KanMx TUB1+3'UTR::HIS3p:mTurquoise-TUB1-LEU2
VMB-619	MATa his3Δ1 leu2Δ0 lys2Δ0 ura3Δ0 Spc110::SPC110-S11A-S36A-sfGFP-CaUra3 Cdc14::Cdc14-tagRFPT-KanMx TUB1+3'UTR::HIS3p:mTurquoise-TUB1-LEU2
VMB-621	MATa his3Δ1 leu2Δ0 lys2Δ0 ura3Δ0 Spc110::SPC110-sfGFP-CaUra3 TUB1+3'UTR::HIS3p:mRuby2-TUB1-LEU2 Clb2::CLB2-3xV5-HphMx
VMB-622	MATa his3Δ1 leu2Δ0 lys2Δ0 ura3Δ0 Spc110::SPC110-S11A-S36A-sfGFP-CaUra3 TUB1+3'UTR::HIS3p:mRuby2-TUB1-LEU2 Clb2::CLB2-3xV5-HphMx

Table S2. Plasmids used in this study

Plasmid	Description	Reference
pMTMA	RITE (Loxp-Flag-hphMx-Loxp-V5)	This study
pBS1539	TAP-tagging at the C-t (Ura3 marker)	(Puig et al., 2001)
p44873	pFA6a-link-yoSuperfolderGFP-CaURA3	(Lee et al., 2013)
p44906	pFA6a-yoTagRFP-T-Kan	(Lee et al., 2013)
p50645	pHIS3p:mRuby2-Tub1+3'UTR::LEU2	(Markus et al., 2015)
p50641	pHIS3p:mTurquoise2-Tub1+3'UTR::LEU2	(Markus et al., 2015)
pNX3b-PK3	pFA6a-PK3-hygMX6	(Amelina et al., 2016)
pRA66	GAL1 driven Cas9	(Anand et al., 2017)

Amelina, H., Moiseeva, V., Collopy, L. C., Pearson, S. R., Armstrong, C. A. and Tomita, K. (2016). Sequential and counter-selectable cassettes for fission yeast. *BMC Biotechnol.* **16**, 1–15.

Anand, R., Beach, A., Li, K. and Haber, J. (2017). Rad51-mediated double-strand break repair and mismatch correction of divergent substrates. *Nature*. **544**, 377–380.

Lee, S., Lim, W. A. and Thorn, K. S. (2013). Improved Blue, Green, and Red Fluorescent Protein Tagging Vectors for S. cerevisiae. *PLoS One*. **8**, e67902.

Markus, S. M., Omer, S., Baranowski, K. and Lee, W. L. (2015). Improved Plasmids for Fluorescent Protein Tagging of Microtubules in Saccharomyces cerevisiae. *Traffic*. **16**, 773–786.

Puig, O., Caspary, F., Rigaut, G., Rutz, B., Bouveret, E., Bragado-Nilsson, E., Wilm, M. and Séraphin, B. (2001). The tandem affinity purification (TAP) method: a general procedure of protein complex purification. *Methods* **24**, 218–229.

Table S3. Primers used in this study

Primer name	Sequence
SPC97-TAP F	TATAGTACCTCCTCGCTCAGCATCTGCTTCTTCCCAAAGAT CCATGGAAAAGAGAAG
SPC97-TAP R	CAAGTTGGTGCACGTCGTTAGTGACATAACGCGTTCATACG ACTCACTATAGGG
SPC110-RITE F	AAGAGATAGAATTGAGAGTAGCAGCGGGCGTATATCTTGG GGTGGATCTGGTGGATCT
SPC110-RITE R	CGATGTACATACGAGAAATATGATGATAGAGTAAGCGATA TGATTACGCCAAGCTCG
SPC110-sfGFP F	AAGAGATAGAATTGAGAGTAGCAGCGGGCGTATATCTTGG GGTGACGGTGCTGGTTTA
SPC110-sfGFP R	CGATGTACATACGAGAAATATGATGATAGAGTAAGCGATA TCGATGAATTCGAGCTCG
SPC42-TagRFP-T F	ATGTCAGAAACATTCGCAACTCCCACTCCCAATAATCGAG GTGACGGTGCTGGTTTA
SPC42-TagRFP-T R	TTTAAGAATGCGCCATACTCCTTAACTGCTTTTTAAATCAT CGATGAATTCGAGCTCG
SPC110 gRNA PAM44 F	Phospho-AAATGGGAGCTTGAAGAACAGTTTT
SPC110 gRNA PAM44 R	Phospho-TGTTCTTCAAGCTCCCATTTGATCA
SPC110 gRNA PAM107 F	Phospho-CGATACAACTTGTGTTTGCGGTTTT
SPC110 gRNA PAM107 R	Phospho-CGCAAACACAAGTTGTATCGGATCA
Donor SPC110 S11A F	AACACTCATGGACGAAGCGTCACATCTCCCAAATGGGGCC TTGAAGAACATGGAATTTACGCCTGTAGGATTTATCAAAT
Donor SPC110 S11A R	ATTTGATAAATCCTACAGGCGTAAATTCCATGTTCTTCAAG GCCCCATTTGGGAGATGTGACGCTTCGTCCATGAGTGTT
Donor SPC110 S36A F	ATTTACGCCTGTAGGATTTATCAAATCCAAGCGAAACACTA CGCAAACACAAGTTGTAGCACCTACTAAGGTTCCAAATGC CAATAATGGTGATGAGAA
Donor SPC110 S36A R	TTCTCATCACCATTATTGGCATTTGGAACCTTAGTAGGTGC TACAACTTGTGTTTGCGTAGTGTTTCGCTTGGATTTGATAA ATCCTACAGGCGTAAAT
Cdc14-TagRFP-T F	CGCCGGTGGTATAAGAAAAATAAGTGGCTCCATCAAGAAA GGTGACGGTGCTGGTTTA
Cdc14-TagRFP-T R	TTTTATTATATGATATATATATATATAAAAATGAAATAAAT CGATGAATTCGAGCTCG
Clb2-V5 F	GGTTAGAAAAAACGGCTATGATATAATGACCTTGCATGAA CGGATCCCCGGGTTAATTAA
Clb2-V5 R	TTATCGTTTTAGATATTTTAAGCATCTGCCCCTCTTCTCAGA ATTCGAGCTCGTTTAAAC

Biology Open • Supplementary information
